# Identification of *CTHRC1* as a novel candidate for neurodevelopmental disorders

**DOI:** 10.3389/fnagi.2026.1737003

**Published:** 2026-01-22

**Authors:** Jie Xu, Yuan He, Zhao Li, Wenrong Zhou, Chunjian Huang, Lu Lu, Akhilesh K. Bajpai, Min Li

**Affiliations:** 1 School of Computer Science and Engineering, Central South University, Changsha, China; 2 CIR Biotech (Shanghai) Co., Ltd., Shanghai, China; 3 In Vitro Biology Unit, WuXi AppTec (Shanghai) Co., Ltd., Shanghai, China; 4 Department of Genetics, Genomics and Informatics, University of Tennessee Health Science Center, Memphis, TN, United States

**Keywords:** Alzheimer’s disease, BXD, cognition, hippocampus, neurodegeneration, systems genetics

## Abstract

**Background:**

Cognitive dysfunction affects over 50 million individuals worldwide, with Alzheimer’s disease (AD) representing two-thirds of cases. We identified *CTHRC1* (Collagen Triple Helix Repeat Containing 1) as a novel candidate associated with cognitive function and neurodegeneration.

**Methods:**

Human proteomic analysis revealed *CTHRC1* as highly upregulated in AD patients (~5-fold increase, adj. *p* = 0.05), with corresponding elevation in 5xFAD mice. Single-cell RNA sequencing showed predominant astrocyte and oligodendrocyte progenitor expression. Using BXD mice, systems genetics analysis revealed associations between hippocampal *CTHRC1* expression and 22 cognition-related phenotypes. PheWAS, ePheWAS, and GWAS analyses confirmed links to nervous system and AD-related traits.

**Results:**

eQTL mapping identified *CTHRC1* as cis-regulated in hippocampus, and correlating with protein transport, transcription, and neurodegeneration pathways. Network analysis revealed 17 direct interactors, including key neurodegeneration genes (BACE1, NEFL, IRS1, VDAC1, SNCAIP) connecting *CTHRC1* to core AD pathways (APP, MAPT, APOE, PSEN1/2). *CTHRC1* overexpression in SH-SY5Y cells promoted tau degradation and modulated network partner expression.

**Conclusion:**

*CTHRC1* represents a central hub in cognitive function networks, suggesting therapeutic potential for neurodegenerative disorders.

## 1 Introduction

Cognitive dysfunction, ranging from mild cognitive impairment to dementia, is associated with higher disability risk and increased health expenditures. Dementia is the most devastating condition affecting today’s elderly population and the leading cause of disability worldwide (Calia et al., 2019; Qin et al., 2022). Over 50 million individuals suffered from dementia in 2019, projected to increase three-fold to 150 million by 2050 (Nichols et al., 2022). Dementia incidence increases exponentially with age, particularly between 65–90 years, doubling approximately every 5 years (Corrada et al., 2010; Jorm and Jolley, 1998). Alzheimer’s disease (AD) accounts for at least two-thirds of dementia cases in people ≥65 years and is the sixth leading cause of death in the United States (Kumar et al., 2025). Hippocampal damage produces inflexible behavior, affecting memory, navigation, exploration, imagination, creativity, and decision-making, as the hippocampus and its interconnected systems support flexible information use (Rubin et al., 2014). Age-related hippocampal alterations, including neuroinflammation from oxidative stress, reduced neurogenesis, and decreased synaptic plasticity, contribute to cognitive decline (Bettio et al., 2017). Several studies have investigated genes associated with cognition and AD (Liu et al., 2021; Hill and Gammie, 2022; Fan et al., 2019), identifying dozens, including APOE (Gibbons et al., 2011; Lefterov et al., 2019), DLGAP2 (Ouellette et al., 2020), APP, PSEN1, PSEN2 (Cruchaga et al., 2012), and STAT3 (Hong et al., 2020). However, due to cognitive dysfunction’s complexity, pathogenic mechanisms remain incompletely understood. Therefore, identifying novel causative agents and drug targets is needed to provide new approaches for diagnosis, treatment, prevention, and management of cognitive impairment.

Systems genetics is an approach that uses a range of experimental and statistical methods to examine intermediate phenotypes, such as transcript, protein, or metabolite levels, to bridge DNA variations with the traits of interest. Furthermore, this approach considers gene-by-gene and gene-by-environment interactions to understand complex traits (Civelek and Lusis, 2014; Moreno-Moral and Petretto, 2016). Cognitive-related disorders are challenging to study directly in humans due to their complexity, environmental uncontrollability, unavailability of samples, and ethical issues. This limitation is overcome by genetic reference populations (GRPs) with controllable environmental factors that provide a suitable platform for linking genetic, environmental, and gene-by-environmental factors to understand such complex traits (Civelek and Lusis, 2014). The fully inbred BXD mouse panel, generated from the crosses between C57BL/6J (B6) and DBA/2J (D2) strains for more than 20 consecutive generations, serves as a suitable animal model for discovering complex mechanisms underlying various physiological conditions (Ashbrook et al., 2021; Andreux et al., 2012). Currently, this family contains over 200 strains with whole-genome sequence data available for ~150, hundreds of omics datasets generated from various tissues including different brain regions, and thousands of phenome datasets (Ashbrook et al., 2021). This panel has been used to show that pattern separation is heritable in the mouse and to identify mechanisms underlying variation in pattern separation (Dickson and Mittleman, 2022). Additionally, a significant difference in operant performance and learning, including faster reversal learning in D2 compared to B6 mice, has been established (Graybeal et al., 2014). The founder strains differ in several types of complex learning, including contextual fear conditioning (Wehner et al., 1997). Furthermore, B6 shows higher myelin transcript expression compared with D2 and accompanying differences in myelin protein composition and content and white matter conduction velocity. The study indicates that genetic variation in myelin gene expression translates to differences observed in axon conduction speed and possibly in hippocampus-related memory and learning tasks (Goudriaan et al., 2020).

Collagen triple helix repeat containing 1 (*CTHRC1*) encodes a protein that may play a role in the cellular response to arterial injury through vascular remodeling. Mutations in *CTHRC1* have been identified to be associated with Barrett esophagus and esophageal adenocarcinoma (Orloff et al., 2011). Furthermore, this gene has been reported to promote tumor progression by regulating the immunosuppression of the tumor microenvironment (Hu et al., 2023). However, a few recent studies, particularly proteomic analyses, have shown *CTHRC1* protein to be deregulated in AD either in human samples or mouse models (Wang et al., 2020; Bai et al., 2020). In a study by Wang et al., the comparison of cortex and serum samples led to the identification of an AD-correlated protein panel of *CTHRC1*, GFAP, and OLFM3 (Wang et al., 2020). The study by Bai et al. found a consistent increase in the expression of *CTHRC1* protein in 5xFAD animals and a high correlation with amyloid-*β* (Aβ) in AD cases (Bai et al., 2020). Another study by Carlyle et al.^1^ found *CTHRC1* protein to be more abundant in the high pathology dementia-AD group than in the normal group. *CTHRC1* is increasingly emerging as a candidate gene in AD because it is repeatedly detected in unbiased proteomic screens, yet its role in the brain remains essentially unexplored. Its known functions—including ECM remodeling (Duan et al., 2022), regulation of cell migration (Li et al., 2022), modulation of inflammatory pathways (Guo et al., 2021), and interactions with Wnt signaling (Kelley, 2008) —are all processes central to hippocampal plasticity and cognitive function. Its secretion and correlation with amyloid burden further highlight its potential relevance both as a mechanistic contributor and as a biomarker. These properties make *CTHRC1* a biologically plausible and underinvestigated candidate for understanding genetic influences on cognition and AD pathology.

**Figure 1:**
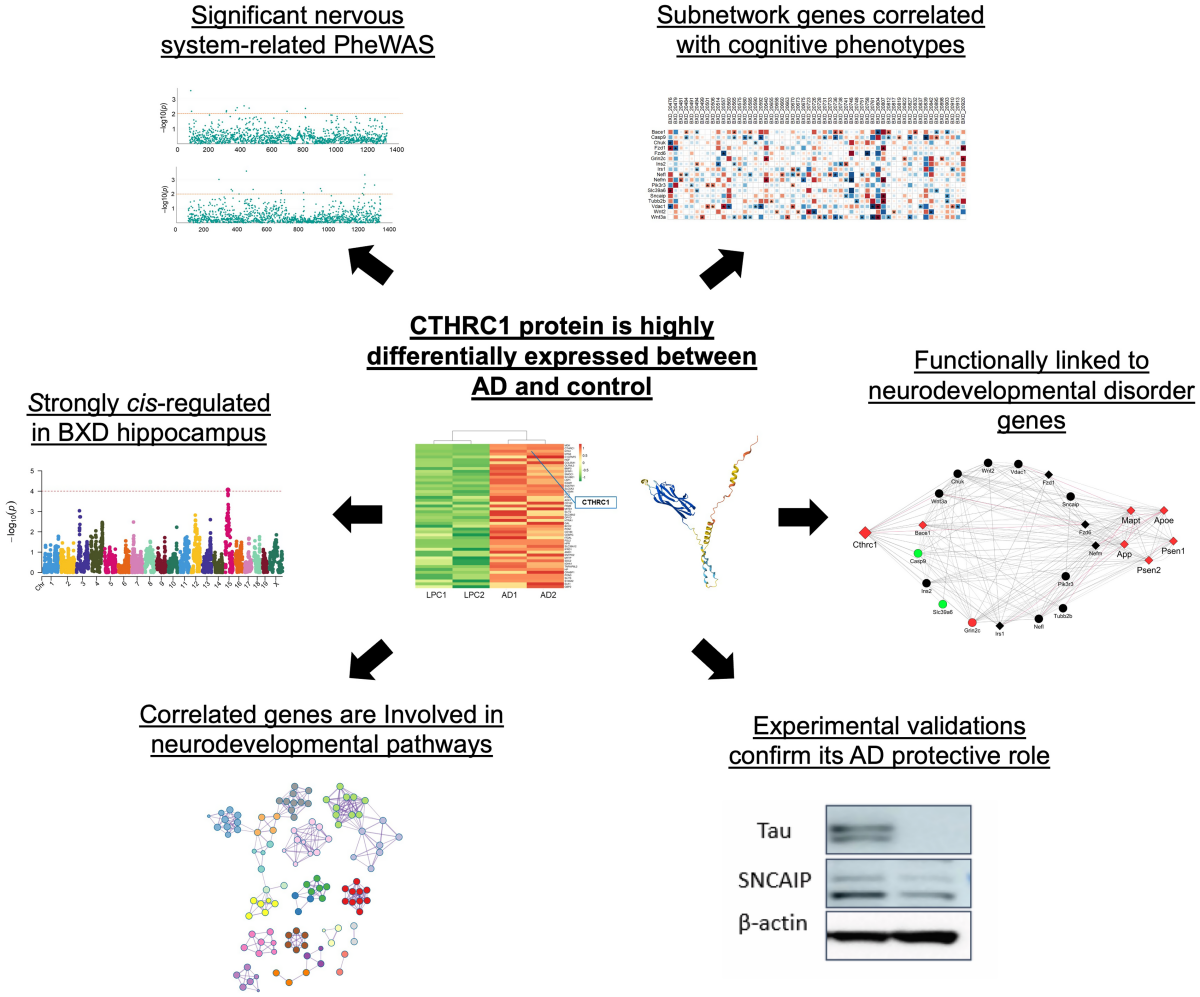
Experimental workflow identifying *CTHRC1* as a candidate gene for neurodegeneration and cognition.

In this study, we used proteomic expression data to identify *CTHRC1* as one of the top differentially expressed proteins in AD patients versus the control group and further validated its expression in 6-month-old 5xFAD mice models. Human genome-wide association data was investigated to link *CTHRC1* mutations with cognition. The hippocampal gene expression data from BXD mice was used for systems genetics analysis to unfold the functional significance of *Cthrc1* and identify its interacting partners. Further, the correlation analysis with learning and memory phenotypes in BXDs was performed to understand the impact of *Cthrc1* expression and that of its interacting partners on cognitive properties. Figure 1 shows a summarized workflow of our study.

## 2 Methodology

### 2.1 Proteomic data analysis

In a recent study, Bai et al. performed deep multilayer proteomics of postmortem brain samples of AD patients and 5xFAD mice models at 3, 6, and 12 months of age using mass spectrometry to identify the molecular network and biomarkers underlying Alzheimer’s disease (Bai et al., 2020). We used the human AD proteomic datasets to identify differentially expressed proteins in the disease state compared to the control samples. The human dataset contained 18 post-mortem samples each for the control and AD group. Each group had two independent pools (9 case per pool). The mean age of the control group was 83 years, whereas that of AD group was 76 years. The 5xFAD mice model (*n* = 4) and wild-type (*n* = 4) samples were then used to confirm the expression difference of the selected genes. Additional details on the samples and their proteomic profiling can be found in the original article (Bai et al., 2020). The differential expression of the proteomics data was performed through the *amica* tool^2^ (Didusch et al., 2022) using the raw intensity values. The *amica* web-tool has been specifically designed for proteomic analysis and uses *limma* R package (Ritchie et al., 2015) to assess the differential expression of proteins.

### 2.2 Hippocampus gene expression dataset

The hippocampus mRNA expression dataset in the current study was generated in our group. The standardized dataset can be accessed through our GeneNetwork portal^3^ by selecting the group as “BXD Family,” type as “Hippocampus mRNA,” and the dataset name as “Hippocampus Consortium M430v2 (June 6) RMA” (GEO Series: GSE84767). A brief description of the experimental protocols used for generating this dataset is provided below, and additional details can be found on our GeneNetwork portal.

### 2.3 Mice and tissue harvesting

The hippocampus data used in the current study was from 71 genetically diverse strains of mice, including 67 BXD recombinant inbred strains, their parental strains (C57BL/6 J, DBA2/J), and two reciprocal F1 hybrids. The majority of the animals were between 45 and 90 days old (mean 66 days, ranging from 41 to 196 days). The mice were obtained from the University of Tennessee Health Science Center (UTHSC), the University of Alabama (UAB), or directly from the Jackson Laboratory. The animals were housed and maintained at UTHSC prior to execution. Animals were deeply anesthetized with 4–5% isoflurane in oxygen until loss of pedal and corneal reflexes, followed by cervical dislocation. Brains were then removed and placed in an RNAlater solution prior to dissection. Cerebella and olfactory bulbs were removed; brains were hemisected, and both hippocampi were dissected whole. All procedures involving mouse tissue were approved by the Institutional Animal Care and Use Committee at the University of Tennessee Health Science Center.

### 2.4 RNA extraction and generation of hippocampus expression data

A pool of dissected tissue, typically from six hippocampi and three naive adults of the same strain, sex, and age, was collected and used to generate cRNA samples. A total of 215 RNA samples were extracted at UTHSC. Briefly, the RNA STAT-60 protocol was used according to the manufacturer’s instructions, which included tissue homogenization, RNA extraction, RNA pretreatment, RNA washing, and RNA purification. Finally, RNA purity was evaluated using the 260/280 nm absorbance ratio, and the samples with values greater than 1.8 were considered. The RNA integrity of the samples was assessed by the Agilent Bioanalyzer 2,100, and those with an RNA integrity number (RIN) greater than 8 were considered for microarray hybridization.

Samples were processed in the INIA Bioanalytical Core at the W. Harry Feinstone Center for Genomic Research, The University of Memphis. RNA samples from 2 to 3 animals of the same age, strain, and sex were hybridized to a single Affymetrix GeneChip Mouse Expression 430 2.0 short oligomer array. The Affymetrix 430v2 arrays consist of ~993,000 25-mer nucleotide probes that can profile the expression of approximately 39,000 transcripts and most known genes and expressed sequence tags. The raw microarray data were normalized using the robust multi-array average (RMA) method (Irizarry et al., 2003) and further converted to an improved z score (2z + 8) (Irizarry et al., 2003). Briefly, in Z-score normalization, instead of leaving the mean at 0 and the standard deviation at 1 unit, we shift up to a mean of 8 units and increase the spread by having a standard deviation of 2 units (2Z + 8 normalized data). This removes the negative values from the tables. Additional details on RNA extraction and hybridization protocols can be found in NCBI-GEO under the accession GSE84767.

### 2.5 Gene–gene and gene-phenotype correlation analyses

Pearson correlation analysis was used to identify *Cthrc1* co-expressed genes in the hippocampus or learning- and memory-related traits associated with the genes being explored. The *R* values with a *p* < 0.05 were considered statistically significant. The mRNA expression values from the “Hippocampus Consortium M430v2 (June 6) RMA” dataset were used for the correlation analysis. The hippocampal gene expression dataset and cognitive phenotype traits can be accessed through our GeneNetwork portal (Mulligan et al., 2017) (see Text footnote 3). The correlation analysis was performed using the *corrplot* v0.92 package^4^ in R.

### 2.6 Phenome-wide association study (PheWAS) and expression-based PheWAS (ePheWAS) analyses

Phenome-wide association studies (PheWAS) have emerged as a viable reverse genetic strategy in humans (Li et al., 2018). Recently this approach has been applied to the BXDs, enabling the discovery of novel gene-phenotype associations. This has been possible owing to the availability of a large amount of genotypic data and thousands of phenotypic traits for BXDs, the largest mouse genetic reference population. Genes that contain high-impact variants, including missense, splice site, and frameshift mutations, as well as genes that have significant cis-e(*p*) QTLs in the BXD transcriptome and proteome datasets, were included in the PheWAS analysis. The association between the genetic variants of each gene, represented by the SNPs, as well as their cis-QTLs were associated with about 5,000 clinical phenotypes. Here, we used a multi-locus mixed-model approach (MLMM) to estimate the associations between each gene and cognitive traits.

In addition, associations between transcript/protein expression and phenotypic traits were estimated using the mixed model regression analysis through ePheWAS. Transcript-trait pairs with fewer than 15 overlapping lines were removed from the analysis. Bonferroni correction was used to perform phenotype-wide significance analysis. The PheWAS and ePheWAS analyses were performed on Systems Genetics at EPFL^5^ (Li et al., 2018), and those with a –log10(*p*) ≥ 2 were considered significant.

### 2.7 Enrichment analysis

The functional enrichment analysis of *Cthrc1*-correlated genes was performed using the Database for Annotation, Visualization and Integrated Discovery (DAVID)^6^ (Sherman et al., 2022) to identify significantly enriched Kyoto Encyclopedia of Genes and Genomes (KEGG) pathways and Gene Ontology biological processes (GO-BP). Mammalian Phenotype Ontology (MPO) analysis was performed using the WEB-based Gene SeT AnaLysis Toolkit (WebGestalt)^7^ (Liao et al., 2019). For this enrichment analysis, “protein coding genes” was selected as the reference set, while ‘minimum number of genes per category’ was kept as 5. The *p*-values were corrected using the Benjamini-Hochberg method for multiple testing, and annotations with an FDR-adjusted *p* < 0.05 were considered statistically significant. Furthermore, the genes were submitted to the Metascape tool^8^ (Zhou et al., 2019) to explore their relationships based on GO-BP terms in the form of a clustered network. The Metascape tool employs a heuristic algorithm to select the most informative terms from the GO clusters. It samples 20 top-score clusters, selects up to the 10 best-scoring terms (lowest *p*-values) within each cluster, then connects all term pairs with Kappa similarity above 0.3.

### 2.8 Functional association network of genes

The *Cthrc1*-coexpressed genes that were involved in the neurodevelopmental pathways were analyzed to predict their functional association network using the GeneMANIA tool^9^ (Warde-Farley et al., 2010). It connects the genes using a very large set of functional association data, including protein and genetic interactions, pathways, co-expression, co-localization, and protein domain similarity. The GeneMANIA cytoscape app^10^ was used for further analysis and visualization of the network.

### 2.9 Collection of genes associated with cognition and neurodevelopmental disorders

The genes important in cognition and AD were collected from multiple gene-function resources using the keywords “cognition” or “Alzheimer.” These keywords were searched in the following databases/repositories: Mouse Genome Informatics (MGI) phenotype/disease association (Human-Mouse: Disease Connection)^11^ (Smith et al., 2018), the NHGRI-EBI GWAS Catalog^12^ (Buniello et al., 2019), and the International Mouse Phenotyping Consortium (IMPC)^13^ (Groza et al., 2023). Translation of human gene lists to mouse orthologs was done using the bioDBnet tool^14^ (Mudunuri et al., 2009).

### 2.10 Brain cell type-specific expression

The brain cell type expression of the genes, including that of *Cthrc1,* was obtained from the Mouse Cell Atlas (MCA)^15^ database (Fei et al., 2022) using the tissue type as “Adult Brain.” The MCA database uses single-cell RNA sequencing to determine the cell type composition of major mouse organs.

### 2.11 *CTHRC1* knockdown and overexpression

The SH-SY5Y human neuroblastoma cell line was obtained from ATCC (CRL-2266). These cells were cultured in DMEM/F12 (1:1) containing 10% fetal bovine serum and maintained at 37 °C in a humidified 5% CO₂ incubator.

*CTHRC1* CRISPR RNA (crRNA) (AUGGCAUUCCGGGU ACACCUGUUUUAGAGCUAUGCU) was synthesized by Azenta. crRNA annealed with tracrRNA (IDT), then incubated with Alt-R^®^ S.p. Cas9 Nuclease V3 (IDT). Generation of a *CTHRC1* knockdown SH-SY5Y cell line was achieved with the Cas9/guide RNA ribonucleoprotein complex (Cas9/RNP) delivered directly to the cells by electroporation. All kits and equipment were provided by Thermo Fisher Scientific. Programmed condition (1,500 V, 20 ms pulse width, 2 pulses) was chosen for electroporation.

A *CTHRC1* wild-type cDNA was cloned in the pLVX-TetOne-Puro vector. SH-SY5Y cells were then infected by the lentivirus encoding full-length *CTHRC1* or empty virus.

### 2.12 Quantitative real-time PCR

The total cellular RNA was extracted by RNeasy^®^ Mini Kit (QIAGEN), and RevertAid First Strand cDNA Synthesis Kit (Thermo Fisher Scientific) was used to reverse transcribe into cDNA. Then real-time quantitative PCR was performed using SYBR^™^ Green PCR Master Mix (Applied Biosystems). Data were analyzed using the 2^-ΔΔCT^ method after standardization with *β*-actin expression levels in each sample. The primers are listed in Supplementary Table 1.

### 2.13 Western blotting

Whole-cell lysates were prepared using RIPA buffer (Sigma), and protein concentrations were quantified with the Pierce BCA Protein Assay Kit (Thermo Fisher Scientific). Equal amounts of protein were resolved on 4–12% Bis-Tris Midi Protein Gels (Thermo Fisher Scientific) and transferred onto membranes using the iBlot^™^ 2 system (Thermo Fisher Scientific). Membranes were blocked with 5% milk for 1 h at room temperature and subsequently incubated with primary antibodies followed by HRP-conjugated secondary antibodies. The following primary antibodies were used: SNCAIP polyclonal antibody (Proteintech, #17818-1-AP; 1:300), Tau (Tau 46) monoclonal antibody (Santa Cruz Biotechnology, #sc-32274; 1:200), and Actin antibody (Cell Signaling Technology, #4967S; 1:1000). Secondary detection was performed using HRP-conjugated anti-rabbit IgG (Cell Signaling Technology, #7074; 1:2000) and HRP-conjugated anti-mouse IgG (Cell Signaling Technology, #7076; 1:2000).

## 3 Results

### 3.1 *CTHRC1* is associated with cognition-related functions and phenotypes

**Figure 2:**
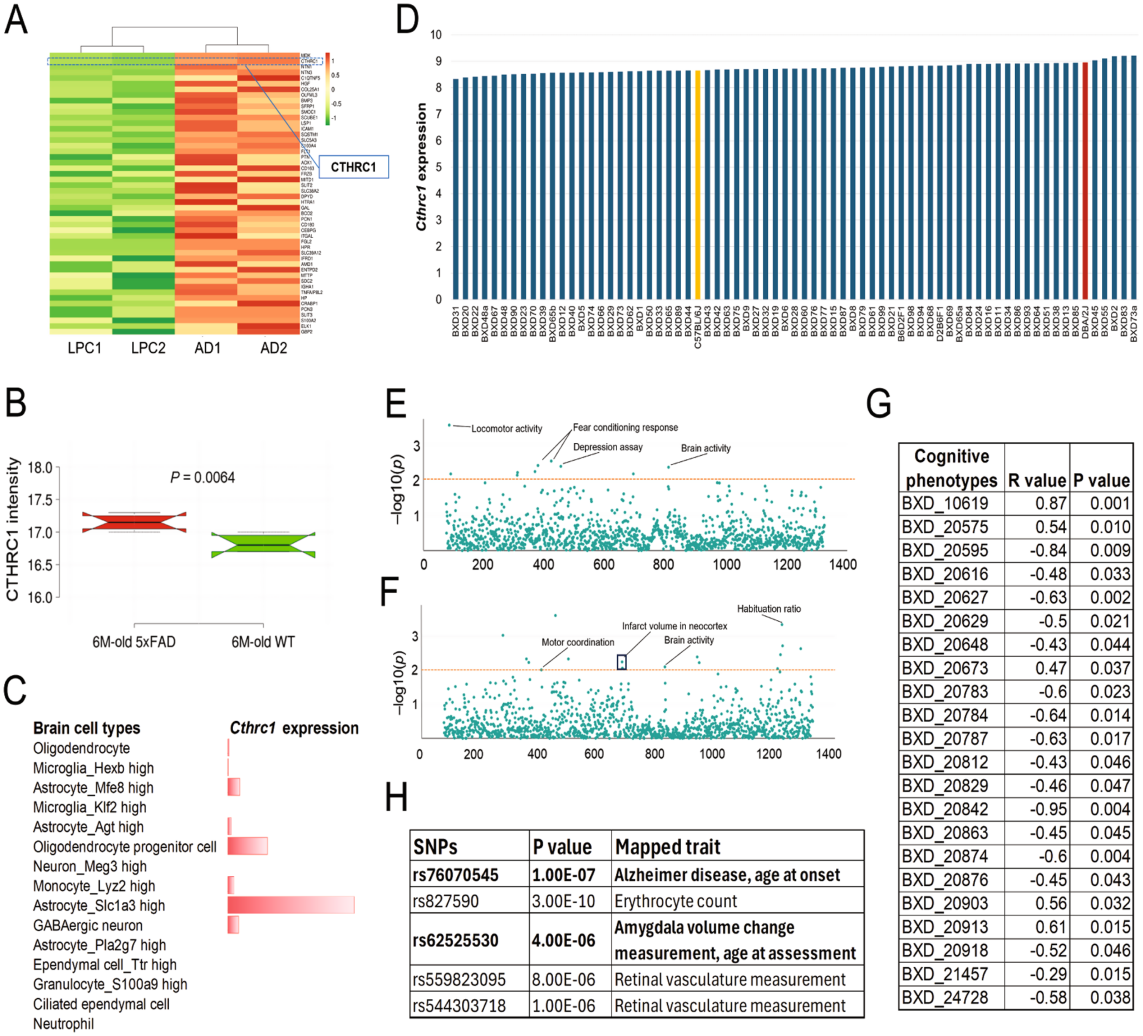
Association of *Cthrc1* with cognition-related properties. **(A)** Top 50 differentially expressed proteins between human AD and control samples. **(B)** Expression of *CTHRC1* protein between 6-month-old 5xFAD and WT mice. **(C)** Expression of *Cthrc1* mRNA in mouse brain cell types based on single-cell RNA sequencing. The scRNA-seq data was collected from the Mouse Cell Atlas (MCA) database (https://bis.zju.edu.cn/MCA/). **(D)** mRNA expression of *Cthrc1* in the hippocampus of 69 BXD strains and two parental strains (B6 and D2). The expression data was obtained from our GeneNetwork database (https://genenetwork.org/). **(E,F)** PheWAS and ePheWAS analysis of *Cthrc1* in BXD mice. Each dot indicates a nervous system phenotype (*x*-axis). The *y*-axis indicates the minus log10(*p*) value. The red dotted line indicates the -log10(*p*) threshold of 2. **(G)** Correlation between *Cthrc1* mRNA levels and learning and memory phenotypes in BXD mice. Both expression data and phenotypes can be accessed from the GeneNetwork database (https://genenetwork.org/) using the phenotype identifier. **(H)** Genomic variants in the human *CTHRC1* locus are associated with different phenotypic traits, including those related to AD. The data is based on Genome-Wide Association Analysis (GWAS, https://www.ebi.ac.uk/gwas/).

The *CTHRC1* gene encodes for collagen triple helix repeat containing 1. Mutations at this locus have been associated with Barrett esophagus and esophageal adenocarcinoma. The differential expression analysis at the protein level between AD and LPC identified *CTHRC1* as the second most upregulated protein based on fold-change. It was found to be upregulated with approximately 5-fold (*adj. p* = 0.05) in AD patients compared to the controls (Figure 2A). This protein also showed a significant increase in its expression in 6-month-old 5xFAD mice compared to their wild-type counterparts (Figure 2B). Furthermore, the cell-type-specific expression of *Cthrc1* based on single-cell RNA sequencing of mouse brain demonstrated its expression predominantly in astrocytes and oligodendrocyte progenitor cells. *Cthrc1* was also found to be expressed to some extent in GABAergic neurons (Figure 2C).

Next, we investigated the mRNA expression of *Cthrc1* in the hippocampus (the brain region primarily associated with learning and memory) of 69 BXD mice and two parental strains (C57BL/6 J or B6, and DBA/2 J or D2) using our GeneNetwork database. The mean expression of *Cthrc1* (probe-set 1452968_at) across BXD strains was found to be 8.7 ± 0.19 SD. BXD31 had the least expression (value = 8.3), whereas BXD73a showed the highest expression (value = 9.2) for *Cthrc1* mRNA with a fold difference of ~1.9 across the BXD strains. Furthermore, we observed a difference of ~1.3 between both the parental strains, with D2 exhibiting higher levels (value = 9.0) of *Cthrc1* than that of B6 (value = 8.7) (Figure 2D). PheWAS analysis using thousands of genomic variants and >5,000 phenotypes from BXD mice suggested significant association of *Cthrc1* SNPs with 10 nervous system-related phenotypes. The phenotypes that were included in the analysis belonged to locomotor activity, depression assay, fear conditioning, and other traits (Figure 2E). Similarly, ePheWAS analysis indicated that *Cthrc1* expression in the BXD hippocampus modulates multiple nervous system-related traits, including phenotypes, such as hippocampus dentate gyrus cells and motor coordination (Figure 2F). When we correlated the hippocampal *Cthrc1* mRNA levels with learning and memory phenotypes (*n* = 341) in BXD mice, the results indicated a significant (*p* < 0.05) correlation of 22 cognition-related phenotypes with *Cthrc1* expression (Figure 2G), suggesting that *Cthrc1* mRNA levels in the BXD hippocampus modulate the phenotypic traits related to cognition. The GWAS results indicated a significant association of variants located in the genomic locus of *CTHRC1* with AD and other brain-related traits, highlighting a strong association of this gene with neurodevelopmental disorders (Figure 2H).

### 3.2 *Cthrc1* expression in the BXD hippocampus is strongly *cis*-regulated

**Figure 3:**
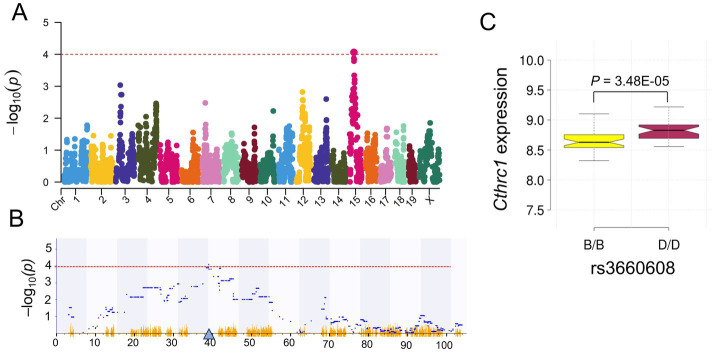
Expression quantitative trait locus (eQTL) mapping for *Cthrc1* expression in the hippocampus of BXD mice. Manhattan plots showing the eQTL **(A)** across the mouse genome and **(B)** on chromosome 15 for *Cthrc1*, identified by the GEMMA mapping method. The *x*-axis indicates the position on the mouse genome in megabases (Mb), whereas the *y*-axis indicates the −log_10_(*p*) score, a value measuring the linkage between gene expression and genomic region. The blue triangle on the *x*-axis indicates the genomic position of the gene. The red horizontal line indicates the significant −log_10_(*p*) score threshold of 4. **(C)** *Cthrc1* is significantly different (*p* = 3.48E-05) between the B and D alleles at 38.99 Mb on Chr 15 (rs3660608).

We performed genome-wide eQTL mapping using the GEMMA mapping method to investigate the genomic regulation of *Cthrc1* mRNA expression. A -log10(*p*-value) of 4 was used as the significance threshold to determine whether *Cthrc1* expression is genomically regulated by epistatic loci. Our results identified mapping of a significant locus for *Cthrc1* on Chr 15 at 38.99 Mb (rs3660608) (Figures 3A,B). The mapped locus was very close to the genomic position of the *Cthrc1* gene (Chr 15 @ 39.08 Mb), indicating that *Cthrc1* mRNA expression in the hippocampus is *cis*-regulated. Thus, *Cthrc1* may genetically regulate other downstream genes in the hippocampus. Statistical analysis of *Cthrc1* expression between the two parental genotypes (B6 and D2) according to the eQTL peak position (rs3660608) showed that BXD mice with the D2 allele exhibit significantly higher expression than those carrying the B6 allele (*p* = 3.48E-05, Figure 3C).

### 3.3 *Cthrc1*-correlated genes in hippocampus modulate neurodevelopmental pathways

**Figure 4:**
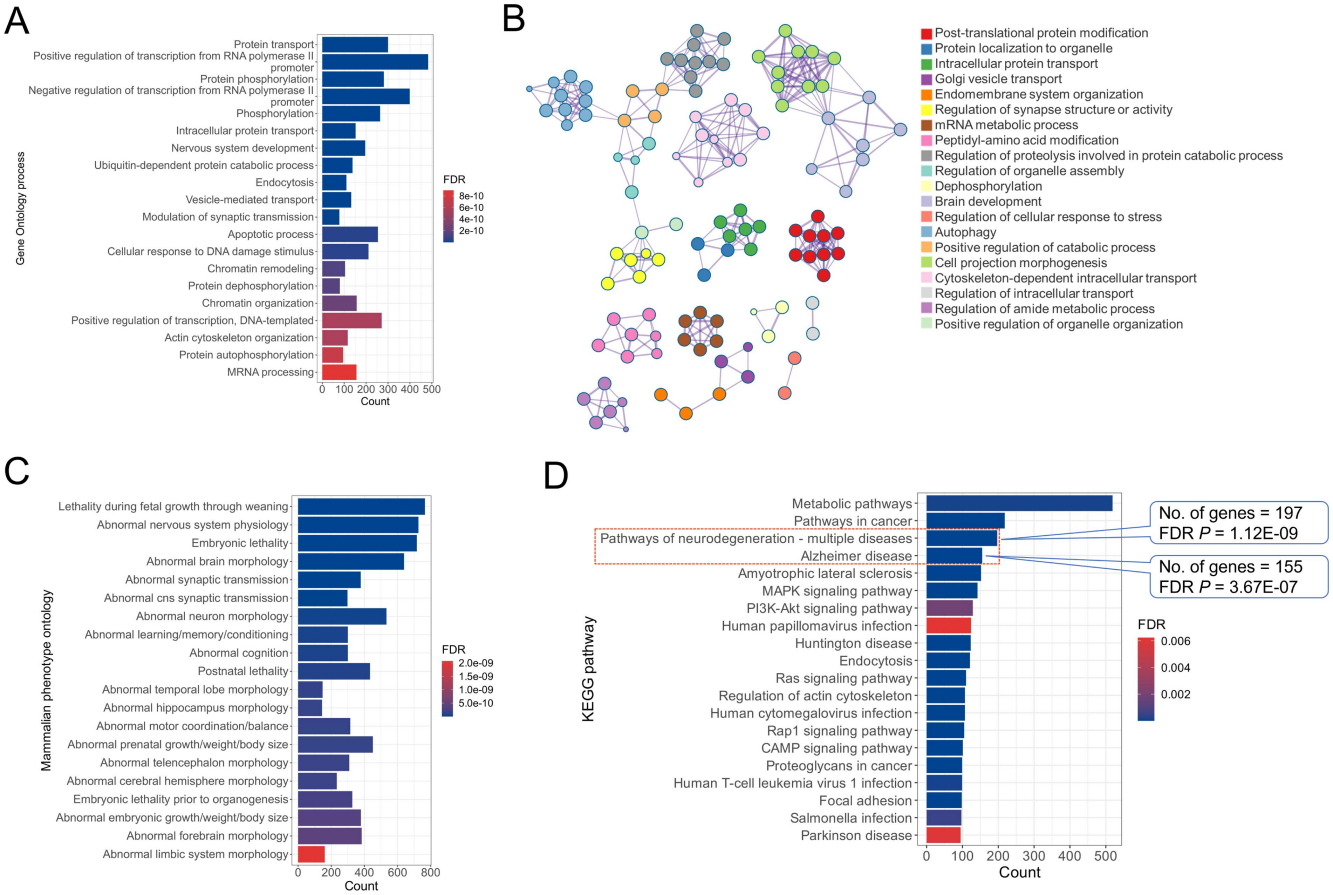
Functional enrichment analysis of *Cthrc1*-correlated genes. **(A)** Top 20 Gene Ontology biological processes (GO-BPs) significantly enriched by *Cthrc1*-correlated genes. **(B)** Top 2000 *Cthrc1*-correlated genes were analyzed using the Metascape tool (https://metascape.org/) to explore the relationships between the GO-BP terms. The nodes represent enriched GO-BP terms and are colored by their respective cluster IDs, whereas the edges link similar terms. The most significant term of the cluster is displayed as a label to represent that cluster. The Metascape tool employs a heuristic algorithm to select the most informative terms from the GO clusters. It samples 20 top-score clusters, selects up to the 10 best-scoring terms (lowest *p*-values) within each cluster, then connects all term pairs with Kappa similarity above 0.3. **(C)** Top 20 mammalian phenotype ontologies and **(D)** Kyoto Encyclopaedia of Genes and Genomes (KEGG) pathways significantly enriched by the *Cthrc1*-correlated genes.

To gain better insights into the pathways and biological functions of *Cthrc1* involved in the hippocampus, we performed a *Pearson* genetic correlation analysis between *Cthrc1* mRNA expression and the entire BXD hippocampal transcriptome data [Hippocampus Consortium M430v2 (June 6) RMA]. At an uncorrected *p*-value of <0.05, 12,475 probes corresponding to 9,305 genes were found to be correlated with *Cthrc1* expression. To further shortlist the genes that are correlated with high confidence, we selected those that had a mean expression of >7 in the BXD hippocampus. This resulted in a total of 9,728 probes corresponding to 7,705 genes. Large-scale transcriptomic datasets commonly yield thousands of correlated genes because hippocampal gene expression is highly modular and dominated by co-regulated networks and shared pathway activity (Oldham et al., 2008). In systems genetics resources such as the BXD panel, the large sample size further increases power to detect broad correlation structures, many of which reflect coordinated biological processes rather than direct regulation (Chesler et al., 2005). Thus, the correlation of *Cthrc1* with such a large number of genes in the hippocampus is implicative of its key role in hippocampal biology and the associated functions. To validate this hypothesis, we performed functional analysis, including KEGG pathway, GO, and MPO enrichment. The results indicated significant representation of cognition-related annotations, further pointing towards the importance of *Cthrc1* in neurodevelopmental disorders. With an FDR *P* cutoff of <0.05, we identified a total of 319 GO-BPs, 300 MPOs, and 170 KEGG pathways. As shown in Figure 4A, “protein transport” was found to be the most enriched GO-BP with 300 genes (FDR *p* = 2.67E-29), followed by “positive regulation of transcription from RNA polymerase II promoter” with 483 genes (FDR *p* = 9.14E-24). The top 50 GO-BPs included several nervous system-related annotations, such as “axon guidance,” “neuron projection development,” “axonogenesis,” and “hippocampus development” (Supplementary File 1). The network of GO-BP terms constructed using the Metascape tool revealed sharing of genes among the annotations related to axon development and hippocampus development. Furthermore, interactions among the terms related to synapse assembly, autophagy, and catabolic processes were also observed. Interestingly, the cluster associated with “cytoskeletal-dependent intracellular transport” formed an independent group and included terms related to synaptic vesicle transport and localization (Figure 4B). Furthermore, the results from the MPO enrichment analysis were found to be in similar lines with that of GO-BPs. Around 15 of the top 20 MPO terms enriched were related to the nervous system (Figure 4C). Some of the important enriched terms included “abnormal nervous system physiology” (*n* genes = 726; FDR *p* = 2.56E-16), “abnormal brain morphology” (*n* genes = 639; FDR *p* = 2.56E-14), “abnormal cognition” (*n* genes = 300; FDR *p* = 4.45E-11), “abnormal hippocampus morphology” (*n* genes = 145; FDR *p* = 1.44E-10), “abnormal learning/memory/conditioning” (*n* genes = 300; FDR *p* = 3.88E-11), and “abnormal neuron morphology” (*n* genes = 533; FDR *p* = 2.56E-11). KEGG pathway analysis demonstrated enrichment of multiple nervous system-related pathways. As shown in Figure 4D, “pathways of neurodegeneration” and “Alzheimer disease” were among the top 5 pathways, based on the percentage of genes involved. While “pathways of neurodegeneration” involved 197 *Cthrc1*-correlated genes, the AD pathway involved 155 genes. Together, both these pathways included 229 *Cthrc1*-correlated genes. The pathway analysis results thus implicate that *Cthrc1* may be linked to the neurodegeneration pathways through these 229 genes. The complete list of significant GO-BPs, MPOs, and KEGG pathways is provided as Supplementary File 1.

### 3.4 Functional gene association network links *Cthrc1* to neurodevelopmental disorder pathways

Together, “pathways of neurodegeneration” and “Alzheimer disease” pathways include 229 *Cthrc1*-correlated genes. Hence, we explored the interaction of these genes with *Cthrc1* using functional association data from GeneMANIA to identify the primary partners of *Cthrc1* and eventually link *Cthrc1* to these pathways. The interaction network (Supplementary Figure 1A) contained 227 query genes (including *Cthrc1*) that were connected with 8,648 edges. The large number of interactions among these genes was expected owing to their involvement in the same pathway. We extracted the *Cthrc1* subnetwork from the large primary network. The subnetwork contained 18 genes (including *Cthrc1*) connected with 97 edges (Supplementary Figure 1B). The subnetwork further revealed that *Cthrc1* physically interacts with *Wnt3a* and *Fzd6* while functionally interacting with the other 15 partners. The nodes BACE1, CASP9, SLC39A6, and GRIN2C were found to be differentially regulated at the protein level in 6-month-old 5xFAD mice compared to their wild-type counterparts. Furthermore, *Fzd1*, *Nefm*, *Fzd6*, *Bace1*, and *Irs1* have been implicated in cognition-related properties or disorders based on collected data from MGI/IMPC/GWAS Catalog. Interestingly, several of these genes in the *Cthrc1* subnetwork were negatively correlated with *Cthrc1* mRNA expression in the BXD hippocampus (Table 1). Next, we wondered if *Cthrc1* is linked to the well-known AD/neurodevelopmental disorder genes (ND genes) through its primary interactors. To this end, we explored the interaction of *Cthrc1*-primary interactors with the well-known ND genes, including *App*, *Mapt*, *Apoe*, *Psen1*, and *Psen2*. Our analysis revealed that several of the *Cthrc1*-primary interactors were directly connected to multiple ND genes. In particular, *Tubb2b*, *Vdac1*, and *Nefm* physically interact with one of the five ND genes. Furthermore, all five ND genes were found to be upregulated in AD compared to the control, either in the 5xFAD mouse model or in human samples at the protein level (Supplementary Figure 1C). Thus, the network analysis results demonstrated that although *Cthrc1* does not directly interact with the ND genes, it affects their function through its primary interacting partners in the same pathway. Furthermore, it is noteworthy that all the ND genes shown in the network were significantly negatively correlated with *Cthrc1* in the BXD hippocampus (Table 1), implying a protective role of *Cthrc1* on neurodevelopmental disorders, such as AD.

**Table 1:** Summary of *Cthrc1*-subnetwork genes.

Gene symbol	*Cthrc1* corr. R	*Cthrc1* corr. R(*p*)	Diff. protein *log2FC* (AD/control)	Diff. protein *p* value (AD/control)	IMPC/MGI/GWAS
Fzd1	0.35	0.0033	*-*	*-*	Y
Wnt2	0.29	0.0152	*-*	*-*	
Vdac1	−0.37	0.0021	*-*	*-*	
Nefm	−0.47	0.00004	*-*	*-*	Y
Fzd6	0.27	0.0254	*-*	*-*	Y
Sncaip	0.35	0.0032	*-*	*-*	
App	−0.26	0.0337	1.05	2.69E-05	Y
Casp9	−0.37	0.0021	−0.48	0.025	
Ins2	−0.25	0.0427	*-*	*-*	
Slc39a6	−0.43	0.0002	−0.24	0.032	
Wnt3a	−0.32	0.0087	*-*	*-*	
Chuk	0.37	0.0019	*-*	*-*	
Bace1	−0.40	0.0008	0.17	0.0019	Y
Mapt	−0.35	0.0034	0.24	0.0168	Y
Irs1	0.38	0.0013	*-*	*-*	Y
Nefl	−0.25	0.0400	*-*	*-*	
Grin2c	−0.28	0.0223	0.14	0.0025	
Psen2	−0.34	0.0052	0.34	0.0002	Y
Tubb2b	−0.27	0.0299	*-*	*-*	
Apoe	−0.28	0.0220	1.00	3.91E-05	Y
Pik3r3	0.25	0.0417	*-*	*-*	
Psen1	−0.28	0.0194	0.23	0.003	Y

The differential expression of proteins was measured in human AD vs LPC or 6-month-old 5xFAD vs wild-type mice.

### 3.5 *Cthrc1*-subnetwork genes are significantly correlated with cognitive phenotypes

**Figure 5:**
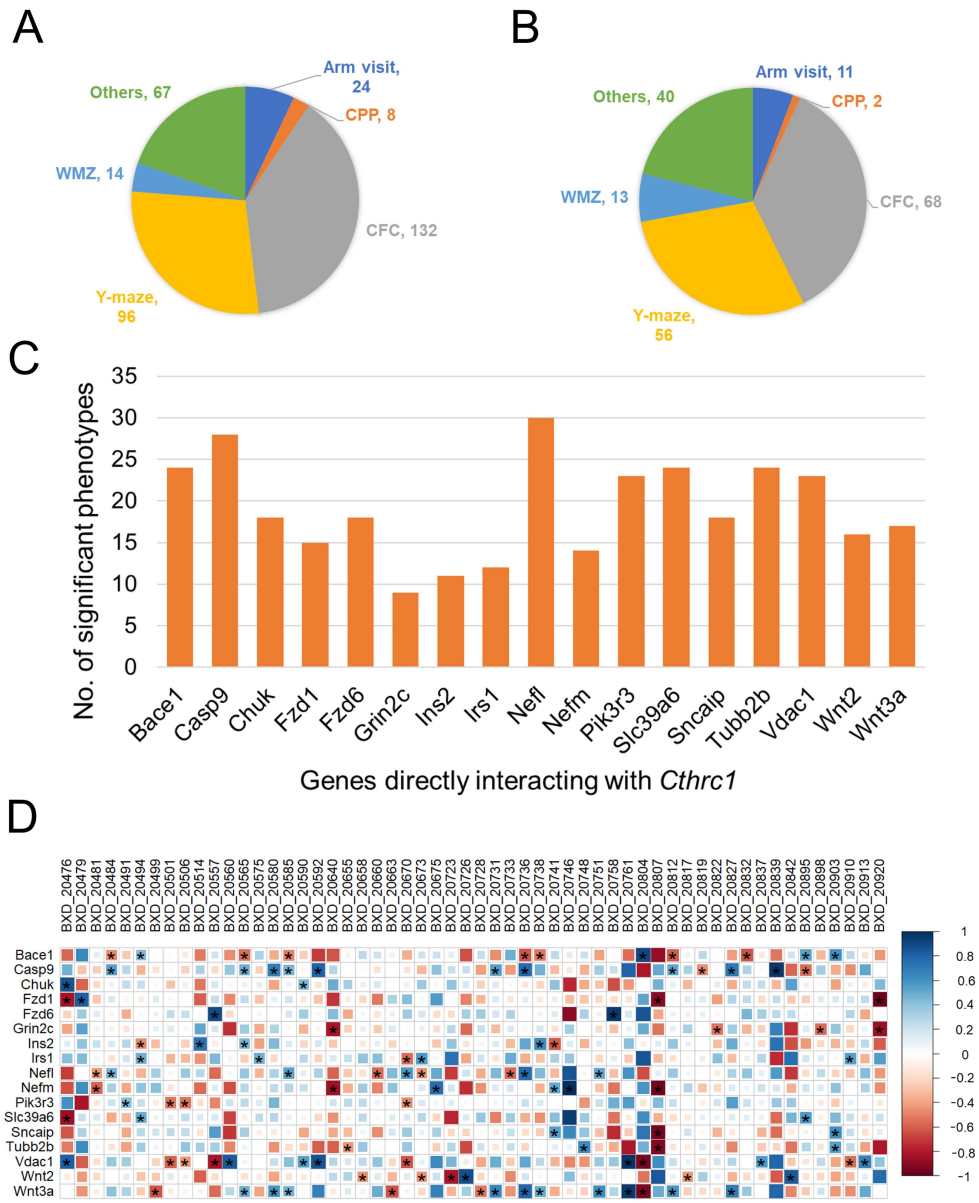
Correlation of learning and memory phenotypes with *Cthrc1*-direct interactors. The hippocampal expression values for the genes interacting directly with *Cthrc1* (*n* = 17) and phenotype trait data (*n* = 341) were obtained from the GeneNetwork portal. The gene-trait analysis was performed using *the corrplot* R package. Number of **(A)** all learning and memory phenotypes and **(B)** significant phenotypes divided into major subgroups. Arm visit: 8-arm radial maze test; CPP: Conditioned place preference; CFC: Contextual fear conditioning; Y-maze: performance on the Y-maze; WMZ: Morris water maze test. The number next to the phenotype group name indicates the number of traits in that group. **(C)** Barplot showing the number of learning and memory phenotypes correlated with each of the 17 genes. **(D)** Correlation plot showing the significance between BXD hippocampal mRNA expression of the 17 genes and Y-maze phenotypes. The *R* values significant with *p* < 0.05 are marked with an asterisk. Blue squares indicate positive correlation, whereas red squares indicate negative correlation. The size and color intensity of the squares are proportional to the correlation coefficient (*R*) values.

While *Cthrc1* is significantly correlated with multiple learning and memory phenotypes from BXD mice (Figure 2G), we intended to explore whether its primary interactors also correlate with the cognitive phenotypes. For the correlation analysis, a total of 341 learning and memory phenotypes belonging to different groups (e.g., Y-maze test, Morris water maze test, and contextual fear conditioning) were retrieved from our GeneNetwork portal. These were then correlated with the hippocampal mRNA expression of the 17 genes that directly interact with *Cthrc1* in the functional association network (Supplementary Figure 1B). Of the total 341 phenotypes (Figure 5A), 190 learning- and memory-related traits were correlated with the hippocampal expression of at least one of the 17 genes (Figure 5B). Among these 190, 36% (*n* = 68) belonged to contextual fear conditioning, whereas 29.5% (*n* = 56) belonged to the y-maze test (Figure 5B). Furthermore, among the 17 genes, *Nefl* correlated with the most number of phenotypes, followed by *Casp9* with 30 and 29 significant correlations, respectively. *Grin2c* correlated with the least number of learning and memory phenotypes (Figure 5C). The Y-maze test is a behavioral task that is used to study spatial learning and memory, which is underlined by the hippocampus (Kraeuter et al., 2019). We (and our collaborators) have collected several traits related to the Y-maze in BXD mice that can be accessed through our GeneNetwork portal. The correlation of the 17 *Cthrc1*-primary interactors with Y-maze phenotype traits indicated significant correlation with 56 of the total 96 traits phenotypes (Figures 5B,D). Of the 17 genes, *Wnt3a* was found to be correlated with the most number of Y-maze-related traits (*n* = 14), followed by *Vdac1* (*n* = 13). Interestingly, *Wnt3a* was found to be physically interacting with *Cthrc1* in the functional association network and was connected to all five ND genes. On the contrary, *Vdac1* was functionally connected to *Cthrc1;* however, it was physically interacting with *microtubule-associated protein tau* (*Mapt*) in the network (Supplementary Figure 1C). The two other genes that correlated with a higher number of Y-maze traits were *Casp9* and *Bace1* (*n* = 12 and 11, respectively). There were seven Y-maze traits, each of which was significantly correlated with 4 *Cthrc1*-primary interactors (Figure 5D; Supplementary File 2).

### 3.6 Genes interacting with *Cthrc1* are expressed in various brain cell types

To explore which brain cell types express *Cthrc1*-interactors, we analyzed the single-cell sequencing data of adult mouse brains from MCA. Our results revealed that many of the genes were predominantly expressed in four brain cell types, *viz.,* astrocyte, microglia, oligodendrocyte, and GABAergic neuron, among the 15 brain cell types investigated. Furthermore, *Bace1*, *Chuk*, *Vdac1*, and *Tubb2b* were found to be expressed in the greatest number of cell types. While the first three genes, *Bace1*, *Chuk*, and *Vdac1,* were expressed in 12 of the 15 brain cell types, *Tubb2b* was found to be expressed in 11 of the 15 brain cell types investigated (Supplementary Figure 2). None of the genes were expressed in neutrophils. The expression of *Tubb2b* was found to be highest in GABAergic neurons, which was also highest among all cell types checked. In addition, it showed high expression in a few other cell types as well, including astrocyte and oligodendrocyte progenitor cells. The expression of *Bace1* was found to be the highest in oligodendrocytes, whereas *Chuk* was found to be highly expressed in neurons. The expression of *Vdac1* was found to be high in ependymal cells and granulocytes. Surprisingly, none of the investigated brain cells expressed *Wnt3a* or *Wnt2* (Supplementary Figure 2).

### 3.7 GWAS analysis indicates association between *Cthrc1*-interactors and neurodevelopmental traits in humans

We used GWASCatalog^16^ to explore the genetic linkage of *Cthrc1*-interacting genes with the neurodevelopmental or cognition related traits in humans. The mutations in 10 of the 17 genes explored were significantly associated with one or more brain related traits in the human population. The following genes were associated with significant cognition or neurodevelopmental related traits: *BACE1*, *FZD1*, *INS*, *IRS1*, *NEFL*, *NEFM*, *PIK3R3*, *SNCAIP,* and *WNT3A*. *SNCAIP* (synuclein alpha interacting protein) showed association with the maximum number of traits (*n* = 12). The traits were mainly related to cognitive performance, Parkinson’s disease, white matter, and neurofibrillary tangles (Supplementary Table 2). The next best gene in terms of the number of associations was *NEFL*, which was correlated with five traits. Both *FZD6* and *CTHRC1* were associated with AD related traits, indicating a close association between both these molecules, which was also evident from the functional association network analysis.

### 3.8 Gene expression changes following *CTHRC1* knockdown or overexpression

We analyzed the changes in the expression of key candidate genes following *CTHRC1* knockdown or overexpression in the SH-SY5Y human neuroblastoma cell line, which is often used as an *in vitro* model for studying neuronal function and differentiation.

**Figure 6:**
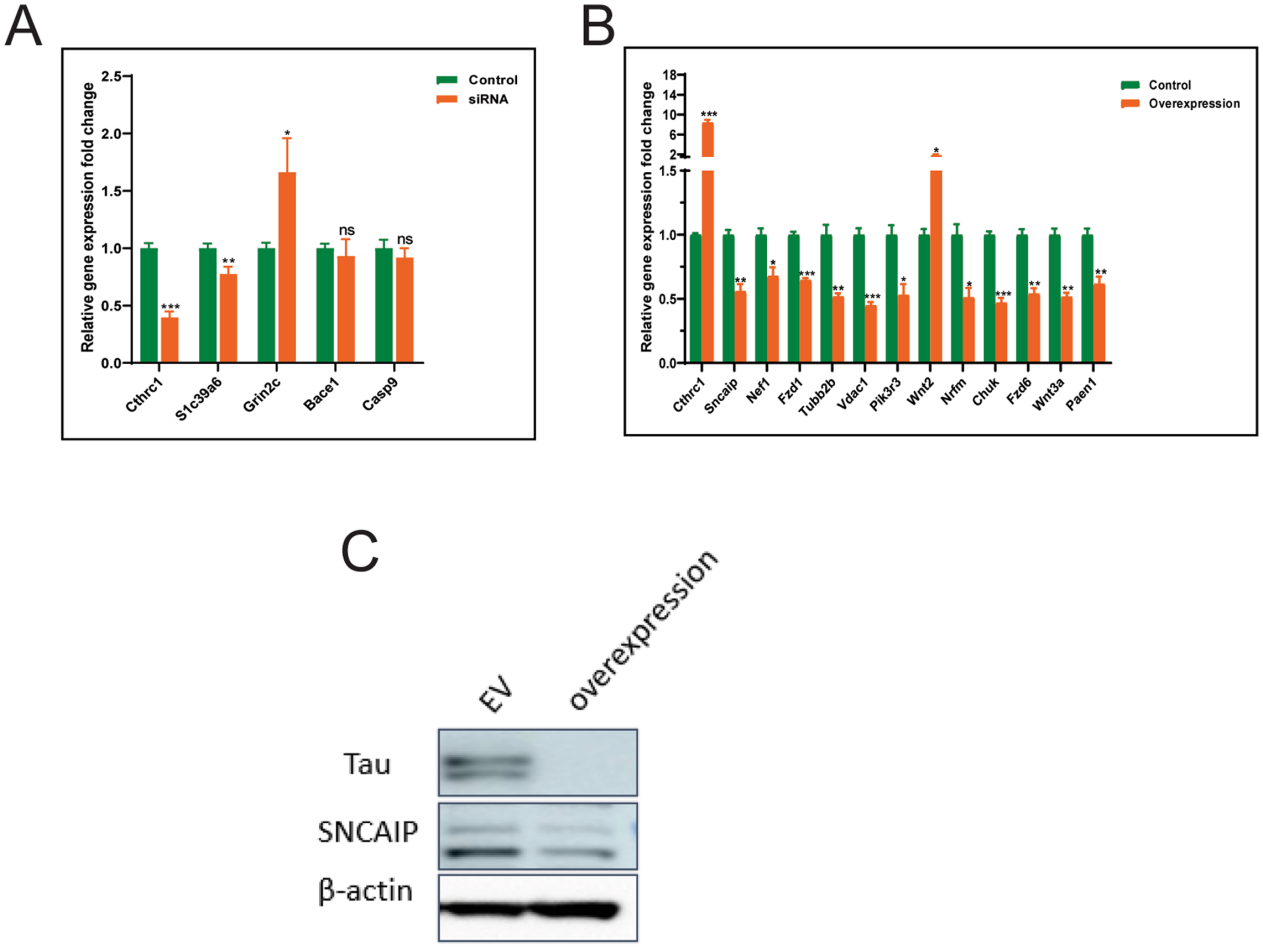
Gene expression changes following *CTHRC1* manipulation in SH-SY5Y human neuroblastoma cells. Relative gene expression changes following *CTHRC1* **(A)** siRNA knockdown or **(B)** overexpression compared to control. Data are presented as mean ± SEM. Statistical significance was determined by *t*-test (*p < 0.05, **p < 0.01, ***p < 0.001; ns, not significant). Green bars represent control, whereas orange bars represent *CTHRC1* knockdown or overexpression. **(C)** Western blotting analysis following *CTHRC1* overexpression in SH-SY5Y cells. *β*-actin was used as a control. EV: empty vector.

Firstly, we used sgRNA technology to knockdown the expression of *CTHRC1* in SH-SY5Y cells using different sgRNA constructs. The preliminary data showed that *CTHRC1*-sgRNA3 could achieve 67% knockdown efficiency; hence, it was used for further knockdown experiments. Following *CTHRC1* knockdown, the expression of SLC39A6 was found to be reduced, while that of GRIN2C was elevated, which was consistent with our *in silico* analysis. However, no significant changes in the expression levels of BACE1 and CASP9 were observed (Figure 6A).

Further, we overexpressed *CTHRC1* in SH-SY5Y cells by using Dox during passages. Interestingly, overexpression of *CTHRC1* led to downregulation of several genes, including SNCAIP, NEF1, FZD1, TUBB2B, VDAC1, PIK3R3, NEFM, CHUK, FZD6, WNT3A, and PSEN1, and upregulation of WNT2 (Figure 6B). Our western blotting data indicated that overexpression of *CTHRC1* in SH-SY5Y cells led to tau degradation. Furthermore, a slight decrease in SNCAIP protein was observed following *CTHRC1* overexpression, pointing out a potential protective role of this gene in AD (Figure 6C).

We constructed an integrated molecular network to visualize how *CTHRC1* may interface with established Alzheimer’s disease pathways (Supplementary Figure 3). The resulting map highlights multiple points of convergence between *CTHRC1*-associated genes and core AD risk factors, including APP, PSEN1, PSEN2, and APOE. Notably, *CTHRC1*-linked signaling modules—including Wnt/Fzd1 activation, MAPT regulation, VDAC1-associated mitochondrial pathways, and SNCAIP/NEFM synaptic components—feed into APP processing and presenilin-dependent cleavage steps. These interactions converge on downstream processes such as amyloidogenic APP processing, tau-related mechanisms, mitochondrial stress, and caspase-mediated injury, collectively pointing toward a multilayered contribution of the *CTHRC1* subnetwork to neurodegenerative outcomes.

## 4 Discussion

In this study, we demonstrate that *Cthrc1* plays an important role in cognitive function. Human proteomic data showed significant upregulation in AD patients compared to controls, which was confirmed at the protein level in 5xFAD mice. PheWAS and ePheWAS analyses linked *Cthrc1* with nervous system phenotypes in BXD mice, while hippocampal *Cthrc1* expression correlated with >20 learning and memory traits. GWAS results indicated *CTHRC1* variants are significantly associated with AD. Using systems genetics and the BXD panel, we established the importance of *Cthrc1* in cognitive function and related disorders.

The hippocampus is central to flexible cognition and social behavior (Rubin et al., 2014; Sweatt, 2004; Anacker and Hen, 2017). Our eQTL mapping revealed that *Cthrc1* is cis-regulated in the BXD hippocampus. Genetic variants located near a gene (cis-eQTLs) typically exert consistent and direct control over that gene’s expression, producing reproducible changes in transcript levels across different tissues, conditions, and populations. This local genetic regulation reflects hardwired genetic control rather than temporary or context-dependent effects increasing the likelihood that cis-regulated genes will have reliable downstream functional consequences (Albert and Kruglyak, 2015; Consortium G.T. et al., 2017; Cookson et al., 2009). The strong cis-eQTL identified for *Cthrc1* indicates that its expression is under stable local genetic control, suggesting that *Cthrc1* may consistently influence downstream regulatory pathways. Pearson correlation analysis identified thousands of genes significantly co-expressed with *Cthrc1*, highlighting its importance in hippocampal biology and cognitive function. Such co-expression analyses are widely used to infer gene function and identify biomarkers, including in neurodevelopmental disorders (Hu et al., 2020; Zang et al., 2023; He et al., 2022).

Functional analysis of *Cthrc1*-correlated genes revealed enrichment in nervous system, cognitive function, and neurodevelopmental disorder related annotations. “Protein transport” was the most significant biological process, involving ~300 genes. Regulated protein transport is essential for synaptic function, requiring dynamic synaptic proteome alterations (Schieweck and Kiebler, 2019). Similar enrichment for protein transport and synthesis has been reported in cognition and neurodevelopmental disorders (Zhang et al., 2018; Rosenberg et al., 2014). Other enriched processes included transcription, protein phosphorylation, and chromatin organization. Altered protein phosphorylation occurs in AD brain (Perluigi et al., 2016), where phosphorylated tau disrupts microtubule stability and cognitive function (Chen et al., 2023; Ferrer et al., 2021). MPO analysis further confirmed strong associations with brain and nervous system terms. KEGG analysis showed “Metabolic pathways” contained the most genes, followed by “Pathways in cancer.” Metabolic dysfunction contributes to neurodevelopmental disorders and AD (Trushina et al., 2013; Yan et al., 2020), as the brain requires substantial energy for neuronal function (Navarro and Boveris, 2010). Age-related glucose utilization decline contributes to cognitive impairment (Petit-Taboue et al., 1998). Cancer pathway enrichment likely reflects *Cthrc1*’s involvement in various cancers (Zhang et al., 2021; Zhao et al., 2022; Mei et al., 2020). The top 5 pathways included “Alzheimer disease” and “pathways of neurodegeneration,” containing 229 genes total, which we explored for interactions with *Cthrc1*.

The interaction network among the 229 ND pathway genes along with *Cthrc1* showed ~8,500 interactions. *Cthrc1*-primary subnetwork contained a total of 17 partners (*Bace1, Casp9, Chuk, Fzd1, Fzd6, Grin2c, Ins2, Irs1, Nefl, Nefm, Pik3r3, Slc39a6, Sncaip, Tubb2b, Vdac1, Wnt2, and Wnt3a*), among which *Wnt3a* and *Fzd6* physically interact with *Cthrc1*. *Wnt3a* hippocampal expression correlated with the maximum number of Y-maze phenotypes in BXD mice, while GWAS revealed its association with brain weight and cortical thickness. The role of Wnt signaling in the development and maintenance of brain structures is widely accepted (Lee et al., 2000; Zhou et al., 2004). *Wnt3a* acts locally to regulate the expansion of the caudomedial cortex, which eventually forms the hippocampus (Lee et al., 2000). Zhou et al. (2004) examined the hippocampal phenotype of single *Lrp6* (a Wnt signaling co-receptor) mutant mice and found that these mice had decreased production of dentate granule neurons and abnormalities of the radial glial scaffolding in the forming dentate gyrus. We could not detect the expression of *Wnt3a* in the adult mouse or human brain, probably because Wnt3a and Wnt2 are predominantly expressed during neurodevelopmental stages and may reflect developmental interactions with *CTHRC1* rather than adult hippocampal expression. Fzd6 correlated with 18 learning/memory phenotypes and acts as a negative regulator of Wnt/*β*-catenin signaling (Pascual-Vargas and Salinas, 2021). Magdesian et al. (2008) reported that Aβ binds to the extracellular cysteine-rich domain of the Frizzled family of proteins and inhibits the Wnt/beta-catenin signaling pathway; blocking of this interaction might prevent neuronal dysfunction in AD. Interestingly, based on our analysis, both *Wnt3a* and *Fzd6* functionally interact with all five ND genes. The other genes that were connected with all five ND genes were *Bace1*, *Chuk*, *Nefl*, *Irs1*, *Grin2c*, *Ins2*, and *Casp9*. *Bace1* and *Grin2c* were found to be upregulated in 6-month-old 5xFAD mice compared to their wild-type counterparts, whereas *Casp9* was found to be downregulated. In addition, *Bace1* (beta secretase 1) and *Irs1* were found to be associated with neurodegenerative disorders or cognition based on data collected by us. GWAS analysis identified *Bace1* variants to be linked with traits, such as apolipoprotein levels and total PHF-tau. Beta-secretase has been reported to participate in the cleavage of the APP protein and promote the cerebral deposition of Aβ, an early and critical feature of AD (Vassar et al., 1999; Hampel et al., 2021). Furthermore, germline and conditional knockout mice for *BACE1* show complex neurological phenotypes (Hampel et al., 2021). These reports were in agreement with the brain cell type expression of *Bace1* in mice, where we detected its expression in multiple cell types, particularly in neurons, oligodendrocytes, and ependymal cells. The ependymal cells are known to be involved in cerebrospinal fluid homeostasis, brain metabolism, and removal of brain waste, and have been implicated in various diseases, including neurodegenerative disorders (MacDonald et al., 2021). Insulin receptor substrate 1 (IRS1), which encodes a protein that is phosphorylated by insulin receptor tyrosine kinase, was another interesting molecule that not only interacted with *Cthrc1* but also all five ND genes. Brain insulin resistance is characteristic of AD and depends on IRS1 phosphorylation (Talbot et al., 2012; Mullins et al., 2017). Our GWAS analysis also linked IRS1 to sTREM-2 and P-tau181p levels in cerebrospinal fluid. Yarchoan et al. demonstrated that abnormal serine phosphorylation of IRS1 is associated with *tau* pathology in AD and tauopathies (Yarchoan et al., 2014). *Nefl* (neurofilament light chain) comprises the exoskeleton that functionally maintains the neuronal caliber. The network showed its physical interaction with APP, in addition to functional association with the other four ND genes. A recent meta-analysis study suggested that increased plasma levels of *Nefl* in AD patients and in patients with mild cognitive impairment (MCI) were associated with cognitive decline (Fan et al., 2023). Another neurofilament protein, *NEFM*, coding for the medium chain, was found to physically interact with two ND genes, *MAPT* and *APOE,* in the functional association network. This gene has been found to be linked to brain morphology traits in humans. *Vdac1* and *Tubb2b* were also physically connected to the ND genes in the functional association network. *Vdac1* encodes a voltage-dependent anion channel, a major component of the outer mitochondrial membrane. It is expressed in most of the brain cell types, with the highest expression in ependymal cells, which are known to be involved in brain metabolism and removal of brain waste. High levels of *VDAC1* have been reported in the brains of post-mortem AD patients and in *APP*-transgenic mice (Shoshan-Barmatz et al., 2018). Smilansky et al. (2015) demonstrated the involvement of *VDAC1* and a *VDAC1* N-terminal peptide (VDAC1-N-Ter) in Aβ cell penetration and cell death induction. The neuronal role of *VDAC1* through maintaining normal mitochondrial homeostasis has been demonstrated by other studies as well (Manczak and Reddy, 2012; Fernandez-Echevarria et al., 2014). In the current study, *Vdac1* significantly correlated with 13 y-maze traits in BXD mice. *Tubb2b* (tubulin beta 2B class IIb), a major component of microtubules, physically interacts with *MAPT* in the functional association network. GABAergic neurons, followed by oligodendrocyte progenitor cells, showed the highest expression for this gene. Using an *APP* knock-in mouse model, Rice et al. (2020) showed that endogenous APP is highly expressed in GABAergic interneurons throughout the hippocampus, suggesting that these cells may have a central role in AD plaque formation, because of which the GABAergic system is considered as a potential therapeutic target for AD (Calvo-Flores Guzman et al., 2018). Mutations in *TUBB2B* are associated with abnormal development of the brain, microcephaly, and axon guidance defects (Jaglin et al., 2009; Romaniello et al., 2012). Another *CTHRC1* interactor, *SNCAIP* (synuclein alpha interacting protein), interacted with *APP* and *MAPT*. It was of particular interest because of its association with a number of cognition- and neurodevelopmental-disorder-related human traits. There is mounting evidence of this gene’s involvement in the second most common type of dementia, the Lewy body diseases (Beyer et al., 2008; Humbert et al., 2007).

Although *CTHRC1* has not yet been identified as a major genome-wide significant AD risk gene, several observations suggest that genetic variation in *CTHRC1* could influence susceptibility to AD or other neurodegenerative diseases. First, *CTHRC1* shows a strong cis-eQTL, indicating that its expression is under stable genetic control, and such regulatory variants frequently contribute to complex trait risk. Second, our integrative analyses show that *CTHRC1* is tightly connected to core AD pathways, including APP/BACE1 processing, PSEN signaling, tau-related mechanisms, mitochondrial function (VDAC1), and inflammatory or synaptic processes. Genes positioned at such network convergence points are often sensitive to regulatory variation that modulates disease outcomes. Third, several *CTHRC1*-interacting partners (APP, PSEN1/2, APOE, MAPT, BACE1, VDAC1) are established AD genes, and variants in upstream modulators of these pathways frequently affect neurodegenerative phenotypes. Together, these findings support the hypothesis that regulatory or coding variants in *CTHRC1* could influence AD-related pathways, thereby modifying risk or progression of AD and potentially other neurodegenerative disorders. Furthermore, the cross-species convergence in our study provides a strong evidence for conserved molecular mechanisms. By observing consistent *CTHRC1*-associated patterns in both human AD datasets and mouse models, our study reduces the likelihood that these findings arise from species-specific artifacts; instead suggests that *CTHRC1* participates in fundamental processes relevant to neurodegeneration and cognition, thereby strengthening the translational value of our results.

We performed experimental validations to verify the results obtained by our *in silico* analysis. *CTHRC1* was knocked down or overexpressed in human neuroblastoma cells, followed by expression profiling of key candidate genes. *CTHRC1* knockdown reduced the expression of SLC39A6, while elevated the expression of GRIN2C. Furthermore, overexpression of *CTHRC1* reduced the expression of several key candidates, including those involved in neurodegeneration, such as PSEN1, SNCAIP, VDAC1, and TUBB2B, and increased the expression of WNT2 (De Strooper and Chavez Gutierrez, 2015; Engelender et al., 1999; Jimenez et al., 2019; Tapia-Rojas and Inestrosa, 2018). Additionally, reduction in tau protein was observed along with a slight decrease in SNCAIP protein. The tau proteins form a group of six highly soluble protein isoforms produced by alternative splicing from the gene MAPT. They have roles primarily in maintaining the stability of microtubules in axons and are abundant in the neurons of the central nervous system (CNS), where the cerebral cortex has the highest abundance. Pathologies and dementias of the nervous system, such as Alzheimer’s disease and Parkinson’s disease, are associated with tau proteins (Chang et al., 2018). The tau hypothesis states that excessive or abnormal phosphorylation of tau results in the transformation of normal adult tau into paired-helical-filament (PHF) tau and neurofibrillary tangles (NFTs). SNCAIP contains several protein–protein interaction domains, including ankyrin-like repeats, a coiled-coil domain, and an ATP/GTP-binding motif. It interacts with alpha-synuclein in neuronal tissue and may play a role in the formation of cytoplasmic inclusions and neurodegeneration (Engelender et al., 1999). These data confirm the protective role of *CTHRC1* in neurodegenerative diseases.

While our study provides evidence supporting the potential role of *CTHRC1* in neurodegenerative processes and cognitive decline, a few limitations should be acknowledged. To clarify the molecular mechanisms underlying *CTHRC1* function, dedicated knockout or overexpression mouse models will be essential. Additionally, further experimental validation is required to define the specific neurodegeneration- and cognition-related pathways influenced by *CTHRC1*. Future studies integrating functional assays, cellular models, and longitudinal *in vivo* analyses will be important for establishing a causal role and delineating its mechanistic contributions of *CTHRC1*.

## 5 Conclusion

Using human genomic/proteomic data and BXD systems genetics analysis, we demonstrated that *Cthrc1* is associated with brain-related phenotypic traits. Functional network analysis identified primary *Cthrc1* interactors, including Bace1, Nefl, Nefm, Irs1, Vdac1, Tubb2b, and Sncaip, which are involved in cognitive functions and neurodegenerative disorders. These molecules connect *Cthrc1* with core neurodegeneration genes (APP, MAPT, APOE, PSEN1/2) in the functional network. Experimental validations using knockdown and overexpression studies confirm the neuroprotective role of *Cthrc1*. Our findings demonstrate for the first time the importance of hippocampal *Cthrc1* in cognitive function and warrant further investigation of its mechanisms in neurodegenerative disorders.

## Data Availability

The original contributions presented in the study are publicly available. The data has been deposited in the GEO repository, accession number GSE84767, https://www.ncbi.nlm.nih.gov/geo/query/acc.cgi?acc=GSE84767.

## Footnotes

1
https://www.biorxiv.org/content/10.1101/2020.07.31.230680v1.full

2
https://bioapps.maxperutzlabs.ac.at/app/amica

3
https://genenetwork.org/

4
https://github.com/taiyun/corrplot

5
https://systems-genetics.org

6
https://david.ncifcrf.gov/

7
http://bioinfo.vanderbilt.edu/webgestalt/

8
http://metascape.org

9
http://www.genemania.org/

10
https://pages.genemania.org/cytoscape-app/

11
https://www.informatics.jax.org/

12
https://www.ebi.ac.uk/gwas/

13
https://www.mousephenotype.org/

14
https://biodbnet-abcc.ncifcrf.gov/db/dbOrtho.php

15
https://bis.zju.edu.cn/MCA/

16
https://www.ebi.ac.uk/gwas/
