# Supplementary material for: Identification of CTHRC1 as a novel candidate for neurodevelopmental disorders
Source: Front Aging Neurosci. 2026 Jan 22;18:1737003. doi: 10.3389/fnagi.2026.1737003 (PMC12872770; doi:10.3389/fnagi.2026.1737003)
Supplement: Supplementary file 1 [file Data_Sheet_1.PDF]

Supplementary material

Identification of CTHRC1 as a novel candidate for neurodevelopmental disorders

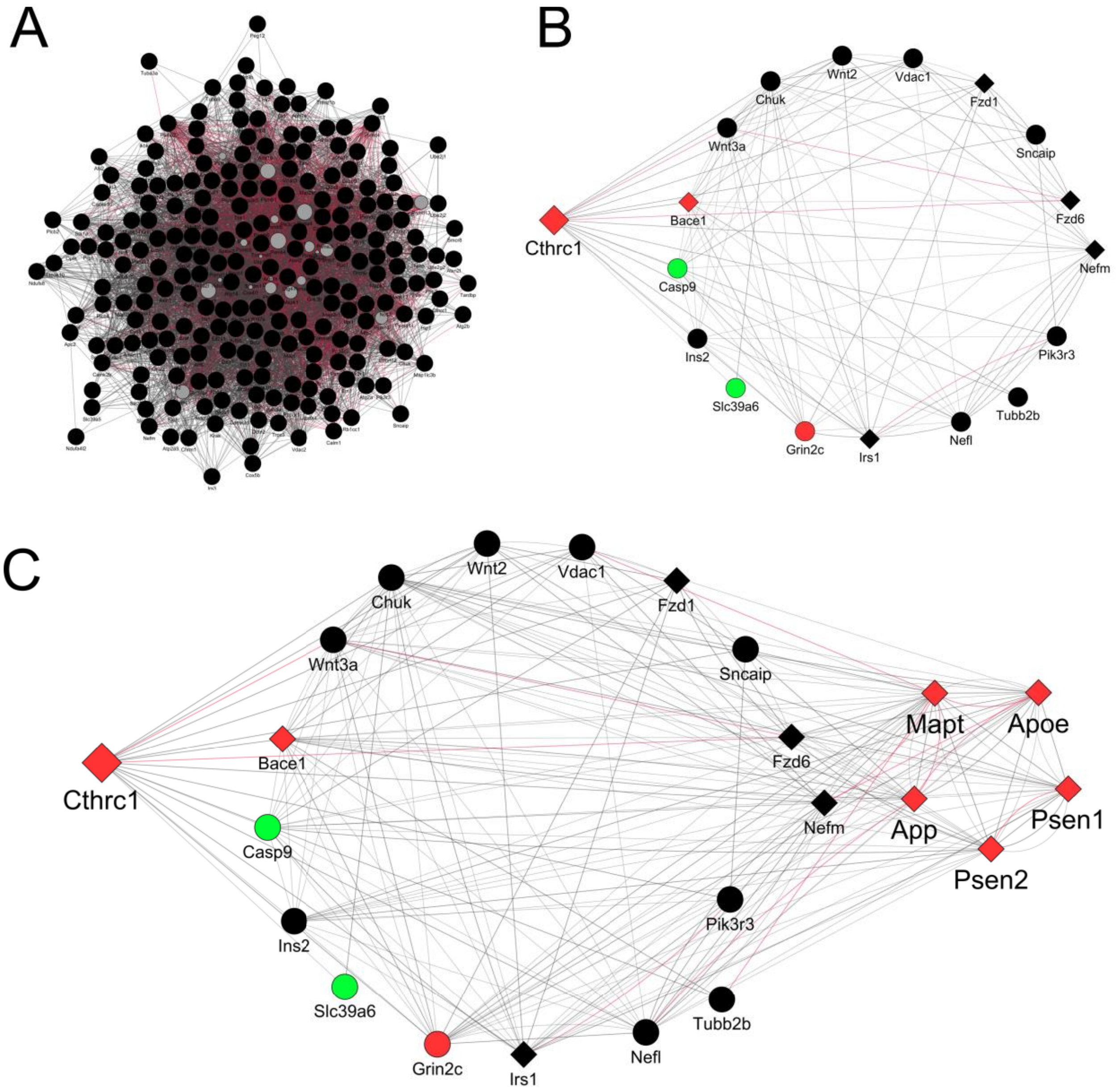

Supplementary Figure 1. Functional association network of neurodevelopmental disorder pathway genes and link to *Cthrc1*. (A) Functional association network of neurodevelopmental pathway disorder genes. The network contains 247 genes connected with approximately 11,000 edges, indicating different functional evidence. **(B)**

Subnetwork of *Cthrc1* interacting partners. The network contains 18 genes and 97 edges. **(C)** Subnetwork containing *Cthrc1* and its link with well-known neurodevelopmental risk genes through *Cthrc1*-interacting partners. Diamond node: Genes related to Alzheimer's disease/cognition based on MGI, IPMC, or GWASCatalog databases; Red nodes: Upregulated in 6-month-old 5xFAD mice vs. WT or human AD vs. LPC; Green nodes: Downregulated in 6-month-old 5xFAD mice vs. WT or human AD vs. LPC; Red lines: Physical interactions.

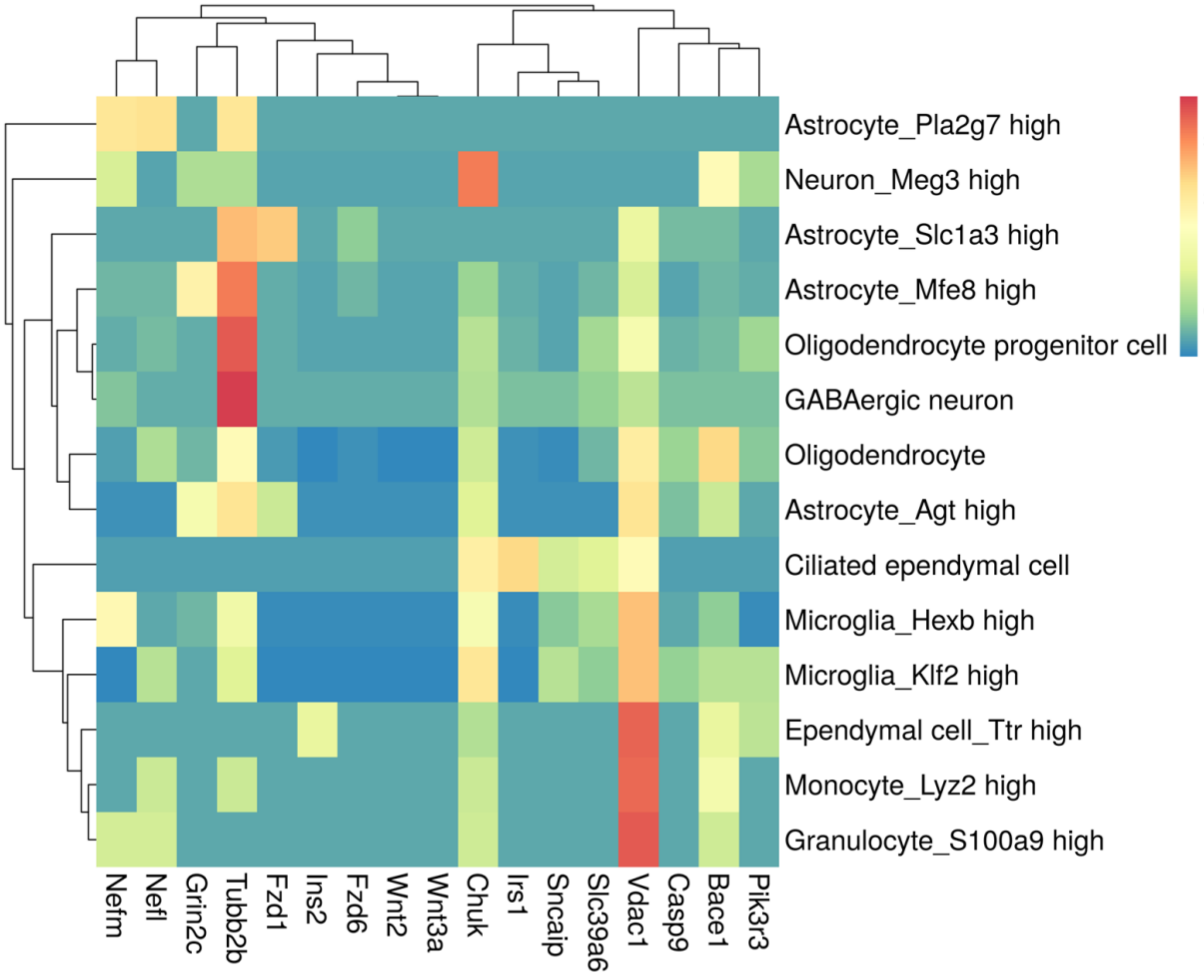

Supplementary Figure 2. Heatmap showing the expression of *Cthrc1*-primary interactors in brain cell types. The columns represent genes, whereas the rows correspond to different cell types. The expression data is based on single-cell sequencing of mouse brain and was obtained from the Mouse Cell Atlas

(https://bis.zju.edu.cn/MCA/search2.html) as *log2*(TPM+1) values. These values were converted to TPM values and then submitted to ClustVis (https://biit.cs.ut.ee/clustvis/) for heatmap construction. Rows were centered; unit variance scaling was applied to rows. Both rows and columns were clustered using correlation distance and the average linkage method.

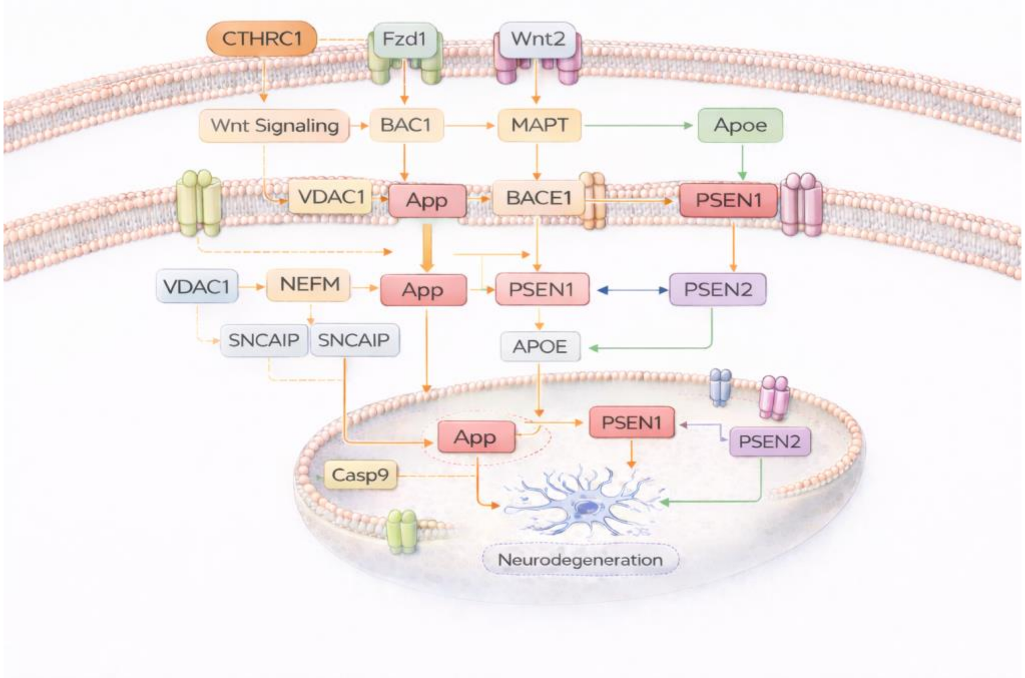

Supplementary Figure 3. **CTHRC1-centered molecular interaction network linking important neurodegeneration related genes.** This schematic illustrates the integrated signaling relationships among CTHRC1 and key Alzheimer's disease–related molecules across plasma and endosomal membranes. CTHRC1 activates the Wnt/Fzd1 pathway, connecting to downstream regulators such as BAC1 and MAPT, while also interacting with VDAC1 and neurofilament components (NEFM). Multiple convergence points center on APP, whose processing is influenced by BACE1 and presenilin complexes (PSEN1 and PSEN2). APOE interacts with MAPT and presenilin-related pathways, further modulating lipid handling and neuroinflammation. SNCAIP, VDAC1, and Casp9 provide additional mechanistic links to synaptic integrity, mitochondrial dysfunction, and apoptotic signaling. Collectively, these interconnected pathways converge on neuronal injury and neurodegeneration.

Supplementary Table 1: Primers used for real-time RT-PCR

Gene	Forward	Reverse
Wnt3a	ATGAACCGCCACAACAACGAGG	GTCCTTGAGGAAGTCACCGATG
Bace1	GTGAGGTTACCAACCAGTCCTTC	CGTGGATGACTGTGAGATGGCA
Casp9	GTTTGAGGACCTTCGACCAGCT	CAACGTACCAGGAGCCACTCTT
Ins2	ACGAGGCTTCTTCTACACACCC	TCCACAATGCCACGCTTCTGCA
Slc39a6	CGTTGGGACTTTGAGTGGTGATG	GACAGATGACTGAAAAGTGGTCC
Grin2c	TGTCATGTGCCTCACTGTGGTG	CACACGGACTTGCCGATAGTGA
Irs1	AGTCTGTCGTCCAGTAGCACCA	ACTGGAGCCATACTCATCCGAG
Nefl	CCAAGACCTCCTCAACGTGAAG	ATGCTTCCCACGCTGGTGAAAC
Tubb2b	GCACGATGGATTCGGTTAGGTC	TCGGCTCCCTCTGTGTAGTGG
Pik3r3	CCACCTAAGCCAATGACTTCAGC	GTTGAGGCATCTCGGACCAAGA
Nefm	ACAACCACGACCTCAGCAGCTA	GTTGAGGAGGTCCTGGTATTCG
Fzd6	GGCAGTGTATCTGAAAGTGCGC	GATGTGGAACCTTTGAGGCTGC
Sncaip	ACACGCAGAGTGTCTACAGCAC	GCAATGGCTTCTGTTTCGCTCAC
Fzd1	GCTTTGTGTCGCTCTTCCGCAT	TACAGCACGCTGAAGACGCCAA
Vdac1	GCAAAATCCCGAGTGACCCAGA	TCCAGGCAAGATTGACAGCGGT
Wnt2	GGAAAGGAAAGGAAAGGATGCCAG	ACAAGAGTGCAAGTGCCACG
Chuk	CTCGGAAACCAGCCTCTCAATG	GATAAACTTCTGGAAGCAAATGGC
Cthrc1	GGGAGGTGGTGGACCTGTATAATG	CACTGCTTGTAGTTGGGTGTCC
Mapt	TCTGATGCTAAGAGCACTCC	CTGTGGTTCCTTCTGGGATC
Apoe	AGCTCAGGGGCCTCTAGAAA	TTGGCCTGGCATCCTGC
Psen1	ACTGTACGTAGCCAGAATGACAA	TACCCTGGGGTCGTCCATTA
App	CTGTGGCAGACTGAACATGC	AGTTCAGGGTAGACTTCTTGGC
Psen2	ATGACAGTTTTGGGGAGCCT	CAAGCTTCACGCCCCTTTC

Supplementary Table 2. GWAS analysis of *Cthrc1*-primary interactors

Gene	Trait	SNPs	P-value
BACE1	Apolipoprotein B levels	rs116987336	2.00E-17
BACE1	Apolipoprotein A1 levels	rs28989504	2.00E-16
BACE1	Apolipoprotein A1 levels	rs1047964	6.00E-30
BACE1	Total PHF-tau (SNP x SNP interaction)	rs6689252, rs477036	9.00E-08
FZD1	Schizophrenia	rs10252923	2.00E-06
INS	Brain morphology (MOSTest)	rs11602347	3.00E-08
FZD6	Alzheimer's disease and age of onset	rs76070545	1.00E-07
FZD6	Amygdala volume change rate x age interaction (1df)	rs62525530	4.00E-06

IRS1	Cerebrospinal fluid sTREM-2 levels	rs2222890	9.00E-06
IRS1	Cerebrospinal P-tau181p levels	rs7558386	1.00E-07
NEFL	Brain morphology (MOSTest)	rs2976442	2.00E-08
NEFL	Brain morphology (MOSTest)	rs2979701	2.00E-08
NEFL	Corpus callosum fractional anisotropy (genu)	rs2979678	1.00E-08
NEFL	Total PHF-tau (SNP x SNP interaction)	rs6879773, rs41414247	8.00E-08
NEFL	Total PHF-tau (SNP x SNP interaction)	rs4907356, rs2976424	8.00E-14
NEFM	Brain morphology (MOSTest)	rs2976442	2.00E-08
NEFM	Brain morphology (MOSTest)	rs2979701	2.00E-08
PIK3R3	Subcortical volume (MOSTest)	rs785482	2.00E-08
PIK3R3	Whole brain restricted isotropic diffusion (multivariate analysis)	rs785485	5.00E-15
PIK3R3	White matter microstructure (mean diffusivities)	rs12029134	4.00E-08
PIK3R3	White matter mean diffusivity x age interaction (2df)	rs68179532	5.00E-08
SNCAIP	Depressive symptoms x independent stressful life events interaction (2df test)	rs2242223	5.00E-06
SNCAIP	Brain shape (segment 1)	rs304391	4.00E-08
SNCAIP	Parkinson's disease progression (motor)	rs5870994	1.00E-06
SNCAIP	Parkinson's disease progression (composite)	rs17367669	3.00E-06
SNCAIP	White matter hyperintensity volume	rs2303655	4.00E-11
SNCAIP	White matter hyperintensity volume	rs2303655	5.00E-11
SNCAIP	White matter hyperintensity volume x hypertension interaction (2df)	rs2303655	8.00E-11
SNCAIP	White matter hyperintensity volume x hypertension interaction (2df)	rs2303655	1.00E-11
SNCAIP	White matter hyperintensity volume (adjusted for hypertension)	rs2303655	4.00E-11
SNCAIP	White matter hyperintensity volume (adjusted for hypertension)	rs2303655	4.00E-11
SNCAIP	Cognitive performance (attention) (longitudinal)	rs138824939	1.00E-05
SNCAIP	Neurofibrillary tangles (SNP x SNP interaction)	rs6883349, rs4857989	5.00E-09
WNT3A	Cortical thickness	rs1745416	4.00E-11
